# Factors associated with considering switching to nicotine pouches among US adults who smoke

**DOI:** 10.1016/j.dadr.2026.100420

**Published:** 2026-03-06

**Authors:** Juhan Lee, Rachel N. Cassidy

**Affiliations:** a Department of Epidemiology and Population Health, Albert Einstein College of Medicine, United States; b Brown University School of Public Health, Center for Alcohol and Addiction Studies, United States

**Keywords:** Nicotine pouches, Harm reduction, Smoking cessation, Epidemiology

## Abstract

**Background:**

Oral nicotine pouches (ONPs) have gained popularity in recent years and are more popular among people who smoke. As ONPs are less harmful than cigarettes, understanding what factors are associated with considering switching to ONPs is critical. This study aims to examine the factors associated with consideration of switching from smoking to ONPs among US adults who smoke.

**Methods:**

Using data from the Wave 7 (2022–2023) of the Population Assessment of Tobacco and Health (PATH) Study (N = 6315), we analyzed a sample of adults who currently smoke. The outcome was whether participants had considered switching from smoking to nicotine pouches, and *a priori* predictors were age, sex, race, ethnicity, income, sexual identity, region, internalizing/externalizing tendencies, current use of e-cigarettes, smokeless/snus, nicotine pouches, days of cigarette smoking, and having tried to quit cigarette smoking.

**Results:**

Among adults who currently smoke in Wave 7 (N = 6315), 122 (weighted %=1.7) reported having considered switching from smoking to ONPs (outcome). In the adjusted model, considering switching was more likely to be associated with being male (vs. female) (aOR=2.75, 95%CI=1.44–5.24), non-Hispanic (vs. Hispanic) (aOR=3.33, 95%CI=1.27–8.70), sexual minorities (vs. heterosexual) (aOR=2.54, 95%CI=1.08–5.99), currently using nicotine pouches (vs. no) (aOR=30.50, 95%CI=13.08–71.10), and having attempted to quit cigarette smoking (vs. no) (aOR = 2.10, 95% CI = 1.14–3.89).

**Conclusions:**

This study identified factors associated with having considered switching to ONPs among U.S. adults who smoke. Policymakers and clinicians should also consider these subgroups when developing and implementing ONP-related policies and guidelines.

## Highlights

• Switching from cigarettes to oral nicotine pouches was more considered by males and non-Hispanic.
• Switching from cigarettes to oral nicotine pouches was more considered by sexual minorities and those who used nicotine pouches.
• Switching from cigarettes to oral nicotine pouches was more considered by those who have attempted to quit cigarette smoking.

## 1 Introduction

Oral nicotine pouches (ONPs) are small microfiber bags filled with nicotine powder, frequently with flavorings and places between the lip and gum (Center for Disease Control and Prevention, 2025). ONPs have gained popularity in recent years in the United States, tripling in sales from $327 million in July 2021 to $1046 million in May 2025 (He et al., 2025). ONPs still expose users to nicotine but are less harmful than cigarettes because they are not combusted; this lack of combustion results in the user being exposed to fewer toxicants and carcinogenic compounds such as tar and carbon monoxide (Travis et al., 2024). Indeed, a recent systematic review of seven randomized trials by Heshmati et al. showed that ONP use was associated with reduced levels of daily cigarette consumption among adults who smoke (Heshmati et al., 2025b). In 2025, U.S. Food and Drug Administration (FDA) Center for Tobacco Products extensively reviewed and compared the health risks and benefits and authorized marketing of some ONP brands, Zyn and On! PLUS, citing their lower health risks relative to combustible tobacco products (US Food and Drug Administration, 2025a, US Food and Drug Administration, 2025b).

ONPs are more popular among people who use other forms of tobacco (Palmer et al., 2025), and one of the most commonly reported reasons for their use is to quit or reduce cigarette smoking (Heshmati et al., 2025b, Tosakoon et al., 2023). Among young adults aged 18–34 years in six U.S. cities, the most frequent reason for ONP use was to quit using combusted tobacco (Tosakoon et al., 2023). In the United States 2022–2023, 2.5% of adults who smoked and 3.1% of adults who used e-cigarettes reported having used ONPs to help quit their tobacco use (Lee et al., 2025). However, ONPs are not FDA-approved smoking cessation aids and might have adverse events (e.g., mouth lesions, upset stomach, sore mouth and throat, acute nicotine toxicity) (Dowd et al., 2024, Kent et al., 2024), thus more research is needed to understand their potential role in promoting smoking cessation and safety, particularly in naturalistic settings.

While ONPs may be reduced-harm products compared to combusted tobacco and a harm reduction option for adults who are currently using combustible tobacco products (Fucito et al., 2025), the full benefits are only realized when adults who smoke completely switch from smoking to ONP use. However, the factors associated with considering switching from smoking to ONPs in adults are understudied in real-world settings. Based on the Theory of Planned Behavior (Ajzen, 1991), behavioral intention is a key antecedent factor of actual behavior; i.e., considering switching from smoking to ONP use. While intentions predict use (Ajzen, 1991), it is also the case that mere exposure can change behavior in the absence of a specific intention. For example, research has shown that e-cigarette use can lead to cigarette quit attempts among dual users, even in those who did not plan to use e-cigarettes to quit (Jackson et al., 2020). While such unintended and unplanned tobacco product switching behaviors might not be uncommon from cigarettes to e-cigarettes (Kasza et al., 2022, Kasza et al., 2021, Shiffman et al., 2025), intention to switch from smoking to other tobacco products (herein, ONPs) is still likely a key factor for predicting future ONP use uptake among adults who smoke and who may not yet have tried ONPs.

A previous study analyzed a nationally representative US adult sample from the 2022–2023 Population Assessment of Tobacco and Health (PATH) Study data and observed that males and having used other cessation methods were more likely to have used ONPs for tobacco use cessation (Lee et al., 2025). The current study will expand this work to include adults who smoke who have considered switching to ONPs; they may or may not have already used ONPs, and this consideration may or may not be expressly for the purpose of quitting cigarettes completely or due to health concerns. There may be differences in factors associated with considering switching from smoking to ONP use by history of smoking quit attempts, given that ONPs are commonly perceived and used as a smoking cessation alternative (Tosakoon et al., 2023); thus, past quit attempts might be related to considering switching from smoking to ONPs. Further understanding these factors will fill in the knowledge gap in factors associated with considering switching from smoking to ONP use, especially by certain subgroups. Furthermore, the previous study did not examine sex differences; however, these differences may be important to understand. For example, male sex has been repeatedly observed as a key subgroup for ONP use (Dai and Leventhal, 2024, Delnevo et al., 2025, Palmer et al., 2025), and ONP marketing frequently targets male consumers (Mead-Morse et al., 2025, Ozga et al., 2024). As sex differences in nicotine product use motivations and cessation behaviors are well-documented (Davis et al., 2023, Smith et al., 2016a, Smith et al., 2016b, Verplaetse et al., 2018), it is worthwhile to examine the sex differences in considering switching from smoking to ONP use.

The goal of this paper was to fill in the knowledge gap in factors associated with considering switching from smoking to ONP use, particularly with respect to history of quit attempts and sex. This study used a nationally representative sample of adults who smoke in the U.S. to examine the factors influencing consideration of switching from smoking to ONPs. Understanding the factors associated with considering a switch to ONPs in a large sample can help tobacco regulatory science and clinicians identify and promote strategies for complete switching.

## 2 Methods

### 2.1 Dataset and analytic sample

This study used a public dataset from Wave 7 (2022–2023) of the Population Assessment of Tobacco and Health (PATH) Study, funded by NIH/NIDA and the FDA Center for Tobacco Products (Hyland et al., 2017) The PATH Study uses a multistage, stratified sampling design of noninstitutionalized civilian adults to ensure representation of the U.S. adult population. It assesses tobacco use behaviors along with sociodemographic, psychosocial, and other behavioral factors. Wave 7 is the first to assess nicotine pouch use behaviors; therefore, prospective tests are not yet possible with this data set.

The analytic sample included adults who currently smoke (i.e., adult respondents who smoked every day or some days, or who had smoked any cigarettes in the past 30 days) (N = 6315), all of whom were asked questions pertaining to the main outcome variable described below. This study was exempt from the Institutional Review Board (IRB) approval because it was a secondary data analysis of a publicly available, completed, and de-identified US national dataset. The original IRB approval for data collection was obtained by Westat, which conducted the PATH Study.

### 2.2 Measures

The outcome variable was whether participants had considered switching from cigarette smoking to ONPs (such as Zyn, On!, or Velo). This was assessed with the question, “Have you considered switching from cigarettes to any of the following products? Choose all that apply,” with the relevant survey item labeled ‘Cigarette Smoker—considered Nicotine pouches (such as Zyn, On!, or Velo),” with response options of “yes” and “no”.

The selection of *a priori* predictors was based on previous literature related to ONP use and smoking cessation behaviors (U.S. Department of Health and Human Services., 2020) The predictors included age (35 or older vs. 18–34 years), sex (female, male), race (White, Black, other), ethnicity (non-Hispanic, Hispanic), annual household income (less than $50,000 vs. $50,000 or more), sexual identity (heterosexual vs. lesbian, gay, bisexual, or other), region (northeast, Midwest, south, west), internalizing tendencies (assessed using GAIN-SS: low, moderate, or high), externalizing tendencies (assessed using GAIN-SS: low, moderate, or high), past-30-day e-cigarette use (no, yes), smokeless/snus use (no, yes), nicotine pouch use (no, yes), days of cigarette smoking in the past 30 days (continuous; range 0–30), having tried to quit cigarette smoking in the past 12 months (no, yes), and harm perception on nicotine (assessed as “How harmful do you think is nicotine to health?”; “not at all harmful,” “slightly harmful,” “somewhat harmful,” “very harmful,” “extremely harmful”; treated as a continuous/ordinal variable) (Persoskie et al., 2019).

Existing evidence suggests various sociodemographic factors that are associated with ONP use (Azagba et al., 2025, Dai and Leventhal, 2024, Delnevo et al., 2025, Lee et al., 2025, Palmer et al., 2025, Patel et al., 2023), which supports the hypothesis that the outcome would be positively associated with being a young adult, male, White, non-Hispanic, sexual minorities, having higher income, and living in US South. Psychosocial factors, such as internalizing tendencies, externalizing tendencies, and risk perception, are well-documented factors for general tobacco and nicotine use and cessation (U.S. Department of Health and Human Services., 2020), which might be consistent with ONP use and support the hypothesis that the outcome would be positively associated with higher levels of internalizing/externalizing tendencies and higher risk perception on nicotine use. Other tobacco use and cessation behaviors might be related to switching from smoking to ONP use, given that ONP use was common among people already using other tobacco products (Azagba et al., 2025, Dai and Leventhal, 2024, Palmer et al., 2025) or who tried to quit their tobacco use (Delnevo et al., 2025, Lee et al., 2025). We thus included these factors in the analyses. We hypothesized that the outcome would be positively associated with other tobacco use (e.g., e-cigarettes, smokeless tobacco, already used ONPs), more days of cigarette smoking, and having tried to quit cigarette smoking.

### 2.3 Statistical analysis

Descriptive statistics were calculated for sample characteristics of adults who currently smoke (N = 6315) and then stratified by the outcome variable (whether participants had considered switching from smoking to ONPs). The significance of bivariate associations between each predictor and outcome was tested using Rao–Scott-adjusted Pearson chi-squared tests for categorical variables and Wald-adjusted *t*-tests for continuous variables. A multivariable binomial logistic regression analysis was conducted to examine whether participants had considered switching from smoking to nicotine pouches based on the predictors listed above. Adjusted odds ratios (aORs) and 95% confidence intervals (CIs) were estimated. We stratified the model by having tried to quit cigarette smoking in the past year and sex, respectively, to examine whether differences exist in associated factors by past history of quit attempts or by sex.

We ran several sensitivity analyses. First, we stratified the model by ONP use status (i.e., among adults who currently smoke and did not use ONPs in the past 30 days vs. among adults who currently smoke and used ONPs in the past 30 days) since previous ONP use might significantly be related to consideration of switching from smoking to ONP use. Second, we ran the alternative model with a 3-level variable coded as 0 =did not attempt to quit cigarette smoking, 1 =  attempted to quit cigarette smoking but did not use evidence-based smoking cessation, 2 =  attempted to quit cigarette smoking and used evidence-based smoking cessation treatment. Herein, “evidence-based smoking cessation treatments” included counseling, a telephone help line or quit line, books, pamphlets, videos, a quit tobacco clinic, class, or support group, or an internet or web-based program, a nicotine patch, gum, inhaler, nasal spray or lozenge, Chantix, varenicline, Wellbutrin, Zyban, or bupropion. To reduce the potential bias from low event outcomes and limited sample size, we ran two sensitivity analyses: (1) the same multivariable logistic regression model but only including factors showing p < 0.2 in their bivariate associations for parsimoniousness (Bursac et al., 2008, MICKEY and GREENLAND, 1989, Zhang, 2016), and (2) the multivariable modified Poisson regression model with a robust error variance (Yelland et al., 2011, Zou, 2004)

The PATH Wave 7 cross-sectional sampling weight (R07_A_C07WGT) was applied, with variance estimation conducted using Fay (0.3)-adjusted balanced repeated replication methods. A significance level of *p* < 0.05 (two-tailed) was used, and analyses followed the STROBE (Strengthening the Reporting of Observational Studies in Epidemiology) guidelines. All statistical analyses were conducted using Stata SE 19.5 (College Station, TX, USA).

## 3 Results

Table 1 presents the sample characteristics of adults who currently smoke (N = 6315) stratified by the outcome variable (whether participants had considered switching from smoking to ONPs). In the analytic sample, 27.6% (weighted) were 18–34 years old, 54.5% were males, 15.4% were Black, 15.0% were Hispanic, 64.8% earned less than $50,000 annually, 12.8% identified as sexual minorities, and 40.4% lived in the South. Among adults who currently smoke in Wave 7 (N = 6315), 122 (weighted % = 1.7) reported having considered switching from smoking to nicotine pouches such as Zyn, On!, or Velo.

**Table 1:** Sample characteristics among adults who currently smoke and stratified by having considered switching from cigarette smoking to nicotine pouches.

		**Having considered switching from cigarette smoking to nicotine pouches (such as Zyn, On! or Velo)**		
	**Total**	**No**	**Yes**	**P-value**
	6315 (100)	6193 (98.3)	122 (1.7)	
**Age, years**				< 0.001
35 or more	3844 (72.4)	3791 (98.8)	53 (1.2)	
18–34	2471 (27.6)	2402 (97.0)	69 (3.0)	
**Sex**				< 0.001
Female	3277 (45.5)	3243 (99.1)	34 (0.9)	
Male	3038 (54.5)	2950 (97.6)	88 (2.4)	
**Race**				0.157
White	4426 (73.8)	4335 (98.2)	91 (1.8)	
Black	1243 (15.4)	1222 (98.5)	21 (1.5)	
Others	646 (10.8)	636 (99.1)	10 (0.9)	
**Ethnicity**				0.002
Non-Hispanic	5212 (85.0)	5099 (98.1)	113 (1.9)	
Hispanic	1103 (15.0)	1094 (99.4)	9 (0.6)	
**Annual household income**				0.268
Less than $50,000	4117 (64.8)	4050 (98.4)	67 (1.6)	
More than $50,000	1884 (35.2)	1832 (97.9)	52 (2.1)	
**Sexual identity**				0.007
Heterosexual	5182 (87.2)	5086 (98.5)	96 (1.5)	
LGB+	1028 (12.8)	1003 (96.8)	25 (3.2)	
**Region**				0.306
Northeast	854 (16.9)	839 (98.7)	15 (1.3)	
Midwest	1690 (24.0)	1659 (98.2)	31 (1.8)	
South	2546 (40.4)	2504 (98.5)	42 (1.5)	
West	1225 (18.8)	1191 (97.6)	34 (2.4)	
**Internalizing tendencies**				< 0.001
Low	3115 (53.7)	3078 (99.0)	37 (1.0)	
Moderate	1437 (22.4)	1404 (97.8)	33 (2.2)	
High	1668 (23.9)	1616 (97.1)	52 (2.9)	
**Externalizing tendencies**				< 0.001
Low	4242 (71.4)	4185 (98.8)	57 (1.2)	
Moderate	1611 (23.6)	1561 (97.2)	50 (2.8)	
High	355 (5.0)	340 (95.8)	15 (4.2)	
**Past-30-day e-cigarette use**				< 0.001
No	4473 (75.6)	4410 (98.8)	63 (1.2)	
Yes	1837 (24.4)	1778 (96.8)	59 (3.2)	
**Past-30-day smokeless/snus use**				< 0.001
No	6038 (95.5)	5943 (98.6)	95 (1.4)	
Yes	269 (4.5)	243 (92.2)	26 (7.8)	
**Past-30-day nicotine pouch use**				< 0.001
No	6178 (98.3)	6105 (98.9)	73 (1.1)	
Yes	128 (1.7)	79 (62.9)	49 (37.1)	
**Days of cigarette smoking in the past 30 days**				0.029
Weighted mean (SD) (ranged 0–30)	22.3 (13.9)	22.4 (11.9)	19.1 (14.3)	
**Past-12-month having tried to quit cigarette smoking**				0.001
No	4034 (69.8)	3972 (98.8)	62 (1.2)	
Yes	1870 (30.2)	1817 (97.3)	53 (2.7)	
**Risk perception on nicotine**				0.204
Not at all harmful	199 (2.7)	194 (98.1)	5 (1.9)	
Slightly harmful	455 (7.4)	440 (97.8)	15 (2.2)	
Somewhat harmful	1573 (25.3)	1538 (97.8)	35 (2.2)	
Very harmful	2083 (33.6)	2055 (98.8)	28 (1.2)	
Extremely harmful	1985 (31.0)	1947 (98.3)	38 (1.7)	

unweighted n (weighted %) for categorical variables and weighted mean (standard deviation) for continuous variables are noted

P-value tests were conducted using Rao-Scott adjusted Pearson chi-squared test for categorical variables and Wald-adjusted T-test for continuous variable.

In the bivariate analyses, participants who had considered switching from smoking to ONPs were more likely to be aged 18–34 years (vs. those 35 or older), male (vs. female), non-Hispanic (vs. Hispanic), sexual minorities (vs. heterosexual), past-30-day use of e-cigarette, smokeless/snus, and ONPs. In addition, they were more likely to have higher levels of internalizing and externalizing tendencies, report fewer days of cigarette smoking in the past 30 days, and report having attempted to quit cigarette smoking in the past 12 months (all *p* < 0.05).

Table 2 presents the results of the multivariable binomial logistic regression examining whether participants had considered switching from smoking to ONPs based on *a priori* sociodemographic, psychosocial, and behavioral factors. In these adjusted models, participants who had considered switching from smoking to ONPs were more likely to be male (vs. female) (aOR = 2.75, 95% CI = 1.44–5.24), non-Hispanic (vs. Hispanic) (aOR = 3.33, 95% CI = 1.27–8.70), sexual minorities (vs. heterosexual) (aOR = 2.54, 95% CI = 1.08–5.99), and those who already used ONPs in the past 30 days (vs. no use) (aOR = 30.50, 95% CI = 13.08–71.10). They were also more likely to report having attempted to quit cigarette smoking in the past 12 months (vs. not having attempted) (aOR = 2.10, 95% CI = 1.14–3.89).

**Table 2:** Results of the multivariable binomial logistic regression model on having considered switching from cigarette smoking to nicotine pouches (such as Zyn, On! or Velo) by *a priori* sociodemographic and behavioral predictors, among total adults who currently smoke (N = 6315).

	**Outcome: Having considered switching from cigarette smoking to nicotine pouches (such as Zyn, On! or Velo)**	
	Adjusted Odds Ratio (95% CI)	P-value
**Age, years**		
35 or more	Reference	
18–34	1.51 (0.92, 2.47)	0.098
**Sex**		
Female	Reference	
Male	**2.75 (1.44, 5.24)**	**0.002**
**Race**		
White	Reference	
Black	1.04 (0.49, 2.24)	0.912
Others	0.50 (0.18, 1.41)	0.189
**Ethnicity**		
Non-Hispanic	**3.33 (1.27, 8.70)**	**0.015**
Hispanic	Reference	
**Annual household income**		
Less than $50,000	Reference	
More than $50,000	1.17 (0.64, 2.16)	0.601
**Sexual identity**		
Heterosexual	Reference	
LGB+	**2.54 (1.08 5.99)**	**0.033**
**Region**		
Northeast	Reference	
Midwest	1.23 (0.47, 3.22)	0.674
South	1.12 (0.40, 3.08)	0.832
West	1.47 (0.56, 3.87)	0.434
**Internalizing tendencies**		
Low	Reference	
Moderate	1.80 (0.85, 3.79)	0.121
High	1.92 (0.83, 4.41)	0.124
**Externalizing tendencies**		
Low	Reference	
Moderate	1.16 (0.59, 2.27)	0.666
High	0.99 (0.38, 2.56)	0.987
**Past-30-day e-cigarette use**		
No	Reference	
Yes	1.32 (0.78, 2.25)	0.302
**Past-30-day smokeless/snus use**		
No	Reference	
Yes	1.33 (0.59, 3.03)	0.489
**Past-30-day nicotine pouch use**		
No	Reference	
Yes	**30.50 (13.08, 71.10)**	**< 0.001**
**Days of cigarette smoking in the past 30 days**		
Continuous (ranged 0–30)	1.01 (0.98, 1.03)	0.641
**Past-12-month having tried to quit cigarette smoking**		
No	Reference	
Yes	**2.10 (1.14, 3.89)**	**0.018**
**Harm perception toward nicotine**		
Treated as continuous (from “Not at all harmful” to “Extremely harmful”)	0.96 (0.77, 1.20)	0.750

Table 3 shows the results of the primary model, stratified by sex. Among females who currently smoke (N = 3277), considering switching from smoking to ONPs was associated with past-30-day nicotine pouch use (aOR=33.82, 95% CI=5.90–193.92) and having tried to quit cigarette smoking in the past year (aOR=3.03, 95% CI=1.07–8.57). Among males who currently smoke (N = 3038), the outcome was associated with non-Hispanic ethnicity (aOR=5.20, 95% CI=1.16–22.38), sexual minority identity (aOR=4.63, 95% CI=1.48–14.49), and past-30-day nicotine pouch use (aOR=35.12, 95% CI=12.78–96.50).

**Table 3:** Results of the multivariable binomial logistic regression model on having considered switching from cigarette smoking to nicotine pouches (such as Zyn, On! or Velo) by *a priori* sociodemographic and behavioral predictors, stratified by sex.

	**Outcome: Having considered switching from cigarette smoking to nicotine pouches (such as Zyn, On! or Velo)**			
	**Among females** **(N = 3277)**		**Among males** **(N = 3038)**	
	Adjusted Odds Ratio (95% CI)	P-value	Adjusted Odds Ratio (95% CI)	P-value
**Age, years**				
35 or more	Reference		Reference	
18–34	1.64 (0.70, 3.85)	0.252	1.55 (0.84, 2.88)	0.161
**Race**				
White	Reference		Reference	
Black	1.14 (0.38, 3.39)	0.817	0.99 (0.37, 2.61)	0.982
Others	0.91 (0.15, 5.45)	0.920	0.39 (0.09, 1.79)	0.226
**Ethnicity**				
Non-Hispanic	1.07 (0.26, 4.49)	0.925	**5.20 (1.16, 23.38)**	**0.032**
Hispanic	Reference		Reference	
**Annual household income**				
Less than $50,000	Reference		Reference	
More than $50,000	1.65 (0.57, 4.76)	0.349	1.02 (0.50, 2.09)	0.951
**Sexual identity**				
Heterosexual	Reference		Reference	
LGB+	0.65 (0.16, 2.68)	0.544	**4.63 (1.48, 14.49)**	**0.009**
**Region**				
Northeast	Reference		Reference	
Midwest	1.41 (0.41, 4.83)	0.577	1.14 (0.35, 3.72)	0.825
South	2.11 (0.65, 6.87)	0.214	0.89 (0.25, 3.23)	0.861
West	1.61 (0.43, 6.04)	0.479	1.23 (0.37, 4.07)	0.736
**Internalizing tendencies**				
Low	Reference		Reference	
Moderate	2.07 (0.52, 8.20)	0.295	1.73 (0.76, 3.95)	0.189
High	1.87 (0.50, 6.98)	0.351	2.00 (0.77, 5.20)	0.153
**Externalizing tendencies**				
Low	Reference		Reference	
Moderate	1.89 (0.48, 7.47)	0.358	0.93 (0.45, 1.94)	0.853
High	3.63 (0.50, 26.08)	0.198	0.63 (0.21, 1.91)	0.407
**Past-30-day e-cigarette use**				
No	Reference		Reference	
Yes	0.74 (0.27, 2.02)	0.553	1.56 (0.84, 2.88)	0.155
**Past-30-day smokeless/snus use**				
No	Reference		Reference	
Yes	3.25 (0.57, 18.51)	0.182	1.14 (0.43, 3.01)	0.787
**Past-30-day nicotine pouch use**				
No	Reference		Reference	
Yes	**33.82 (5.90, 193.92)**	**< 0.001**	**35.12 (12.78, 96.50)**	**< 0.001**
**Days of cigarette smoking in the past 30 days**				
Continuous (ranged 0–30)	1.02 (0.98, 1.07)	0.340	1.00 (0.97, 1.03)	0.986
**Past-12-month having tried to quit cigarette smoking**				
No	Reference		Reference	
Yes	**3.03 (1.07, 8.57)**	**0.037**	1.66 (0.83, 3.30)	0.150
**Harm perception toward nicotine**				
Treated as continuous (from “Not at all harmful” to “Extremely harmful”)	1.11 (0.69, 1.79)	0.662	0.93 (0.72, 1.21)	0.599

Table 4 shows the results of the primary model, stratified by past history of smoking quit attempts. Among adults who currently smoke and have not tried to quit cigarette smoking in the past 12 months (N = 4034), the outcome was associated with male gender (aOR=3.48, 95% CI=1.16–10.46) and past-30-day nicotine pouch use (aOR=38.82, 95% CI=13.28–113.48). Among adults who currently smoke and have tried to quit cigarette smoking in the past 12 month (N = 1870), the outcome was associated with male gender (aOR=2.36, 95% CI=1.08–5.17), sexual minority identity (aOR=2.30, 95% CI=1.15–4.62), higher levels of internalizing tendencies (aOR=4.78, 95% CI=1.54–14.86 for moderate level, aOR=3.50, 95% CI=1.08, 11.32 for high level; vs. low) and past-30-day nicotine pouch use (aOR=27.80, 95% CI=7.20–107.27).

**Table 4:** Results of the multivariable binomial logistic regression model on having considered switching from cigarette smoking to nicotine pouches (such as Zyn, On! or Velo) by *a priori* sociodemographic and behavioral predictors, stratified by past-12-month having tried to quit cigarette smoking.

	**Outcome: Having considered switching from cigarette smoking to nicotine pouches (such as Zyn, On! or Velo)**			
	**Among those who have not tried to quit cigarette smoking in the past 12 month** **(N = 4034)**		**Among those who have tried to quit cigarette smoking in the past 12 month** **(N = 1870)**	
	Adjusted Odds Ratio (95% CI)	P-value	Adjusted Odds Ratio (95% CI)	P-value
**Age, years**				
35 or more	Reference		Reference	
18–34	0.94 (0.40, 2.19)	0.876	2.14 (0.99, 4.64)	0.053
**Sex**				
Female	Reference		Reference	
Male	**3.48 (1.16, 10.46)**	**0.026**	**2.36 (1.08, 5.17)**	**0.032**
**Race**				
White	Reference		Reference	
Black	0.85 (0.26, 2.80)	0.790	1.26 (0.48, 3.32)	0.640
Others	0.37 (0.09, 1.48)	0.159	0.58 (0.10, 3.38)	0.543
**Ethnicity**				
Non-Hispanic	2.00 (0.55, 7.21)	0.287	7.13 (0.78, 65.08)	0.081
Hispanic	Reference		Reference	
**Annual household income**				
Less than $50,000	Reference		Reference	
More than $50,000	1.24 (0.55, 2.79)	0.603	1.03 (0.42, 2.49)	0.951
**Sexual identity**				
Heterosexual	Reference		Reference	
LGB+	2.83 (0.47, 16.86)	0.251	**2.30 (1.15, 4.62)**	**0.019**
**Region**				
Northeast	Reference		Reference	
Midwest	1.17 (0.29, 4.66)	0.820	1.42 (0.37, 5.49)	0.611
South	0.49 (0.10, 2.36)	0.372	2.84 (0.70, 11.50)	0.143
West	1.06 (0.28, 4.00)	0.936	2.49 (0.64, 9.78)	0.188
**Internalizing tendencies**				
Low	Reference		Reference	
Moderate	0.88 (0.34, 2.30)	0.789	**4.78 (1.54, 14.86)**	**0.007**
High	1.43 (0.41, 4.99)	0.569	**3.50 (1.08, 11.32)**	**0.037**
**Externalizing tendencies**				
Low	Reference		Reference	
Moderate	0.82 (0.30, 2.24)	0.697	1.65 (0.66, 4.09)	0.279
High	1.25 (0.29, 5.37)	0.765	0.82 (0.16, 4.18)	0.812
**Past-30-day e-cigarette use**				
No	Reference		Reference	
Yes	1.60 (0.86, 2.96)	0.135	1.14 (0.45, 2.90)	0.775
**Past-30-day smokeless/snus use**				
No	Reference		Reference	
Yes	1.06 (0.36, 3.15)	0.919	1.69 (0.44, 6.59)	0.443
**Past-30-day nicotine pouch use**				
No	Reference		Reference	
Yes	**38.82 (13.28, 113.48)**	**< 0.001**	**27.80 (7.20, 107.27)**	**< 0.001**
**Days of cigarette smoking in the past 30 days**				
Continuous (ranged 0–30)	0.99 (0.96, 1.01)	0.305	1.02 (0.99, 1.06)	0.255
**Harm perception toward nicotine**				
Treated as continuous (from “Not at all harmful” to “Extremely harmful”)	0.93 (0.66, 1.30)	0.658	1.05 (0.67, 1.64)	0.826

Supplemental Table 1 shows the Results of the multivariable binomial logistic regression model on having considered switching from smoking to nicotine pouches (such as Zyn, On! or Velo), stratified by past-30-day use of ONPs. Among those with no past-30-day nicotine pouch use, those considering switching from smoking to ONP use were still more likely to be male (aOR=2.74, 95% CI=1.37, 5.48) and to have tried to quit smoking in the past 12 months (aOR=2.30, 95% CI=1.15, 4.60). Among adults who smoke and reported past-30-day nicotine pouch use, there was no significant difference in the outcome by predictors, suggesting that past ONP use appeared to be strongly associated with consideration of switching.

Supplemental Table 2 shows the results of the multivariable binomial logistic regression model on having considered switching from smoking to nicotine pouches (such as Zyn, On! or Velo), with a 3-level variable coded as 0 =did not attempt to quit cigarette smoking, 1 =  attempted to quit cigarette smoking but did not use evidence-based smoking cessation (i.e., NRT, prescribed medications, and behavioral counseling), 2 =  attempted to quit cigarette smoking and used evidence-based smoking cessation treatment. Compared to those who did not try to quit cigarette smoking, those who tried to quit cigarette smoking and used evidence-based smoking cessation strategies were more likely to report considering switching to ONPs (aOR=3.10, 95% CI=1.30, 7.36). There was no significant difference in outcome between no-quit-attempt (0) vs. attempted to quit but did not use evidence-based treatment (1) (p = 0.150), nor attempted to quit but did not use evidence-based treatment (1) vs. attempted to quit and did use evidence-based treatment (2) (p = 0.194).

Supplemental Tables 3 and 4 show the results of two sensitivity analyses: (1) logistic regression model only including factors with p < 0.2 in their bivariate association (i.e., age, sex, race, ethnicity, sexual identity, internalizing/externalizing tendencies, past-30-day use of e-cigarettes, smokeless tobacco, nicotine pouches, days of cigarette smoking, past-12-month having tried to quit cigarette smoking; Supplemental Table 3) and (2) a modified Poisson regression with a robust error variance (Supplemental Table 4). Consistent with the primary model, having considered switching from smoking to ONP use was positively associated with being male, non-Hispanic, sexual minorities, past-30-day nicotine pouch use, and past-12-month having tried to quit cigarette smoking.

## 4 Discussion

This study identified factors associated with having considered switching to ONPs among U.S. adults who smoke. Being male, non-Hispanic, a sexual minority, currently using nicotine pouches, and having attempted to quit cigarette smoking were associated with considering switching to ONPs. Furthermore, we observed differences in associated factors by sex and past history of smoking quit attempt (discussed below), which is a novelty of this study. This study is notable for focusing on a real-world population, which provides insight into how adults who smoke may be making decisions about switching to ONPs in the absence of specific guidance to do so. As ONP use continues to gain popularity, understanding the characteristics of people who smoke and are considering switching is important to inform regulation and clinical practice and to encourage full switching to reduce harm from smoking.

Regarding sociodemographic factors, being male, non-Hispanic, and a sexual minority were more likely to be associated with considering switching to ONPs. Nicotine pouches are well-documented to be more frequently used by males (Dai and Leventhal, 2024, Delnevo et al., 2025, Lee et al., 2025). Our findings align with this observation, suggesting that male adults who smoke may be more interested in switching to ONPs. Indeed, recent research has shown that males who used tobacco products were more likely to report ONP use for their tobacco use cessation (Lee et al., 2025). Further, Hispanic individuals are generally less likely to report tobacco use, including nicotine pouches. Previous studies have shown that nicotine pouch use was generally higher among non-Hispanic individuals (Dai and Leventhal, 2024, Delnevo et al., 2025, Palmer et al., 2025), which aligns with our finding that non-Hispanic adults who smoke may be more interested in switching to ONPs from cigarette smoking. Research on ONP use among adults who smoke and identify as sexual minorities remains limited. A study by Tosakoon et al. found that heterosexual respondents reported higher levels of ONP use (63%) than sexual minorities (37.0%) among young adults; however, this association was no longer significant when controlling for other factors (Tosakoon et al., 2023). More research is needed on ONP use and smoking cessation behaviors among adults who smoke and identify as sexual minorities.

As might be expected, participants who had considered switching to ONPs were more likely to use nicotine pouches and to have previously attempted to quit cigarette smoking. This is an encouraging finding, as fully switching to ONPs and quitting smoking would benefit the health of these individuals. Note that none of the predictors were significantly associated with switching from smoking to ONP use among adults who smoke and had already endorsed using nicotine pouches, suggesting that once someone uses nicotine pouches, this factor is likely to be the most important in considering switching from smoking to ONP use fully, over and above other factors. Motivations for considering switching from smoking to ONPs remain understudied but may include health concerns (e.g., perceiving nicotine pouch use as less harmful than combustible cigarette smoking), exposure to ONP marketing (e.g., messages portraying ONPs as less harmful than cigarettes, or smoking cessation tools), convenience (e.g., the ability to use ONPs in places where cigarette smoking is prohibited), or reduced odor (Long et al., 2023, Sharma et al., 2024, Tosakoon et al., 2023, Zamarripa et al., 2024). Future studies should qualitatively examine the reasons among adults who smoke consider switching from smoking to ONP use and their experiences with doing so (Tosakoon et al., 2023).

We also observed sex differences in factors associated with considering switching to ONPs, finding that considering switching to ONPs was associated with current nicotine pouch use for both males and females, but a greater past history of quit attempts was associated with a greater likelihood of considering switching only for females; and being non-Hispanic and/or a sexual minority was associated with considering switching only for males. Given the gender difference in nicotine and tobacco product use motivations are well documented, such as different nicotine reinforcement (e.g., females were more likely to use tobacco/nicotine to cope with their stress while males for positive nicotine reinforcement) (Davis et al., 2023),our findings might be related to sex differences in unique motivations for ONP use from cigarette smoking. In our findings, females might be more likely to consider switching from smoking to ONP use based on their past history of failed quit attempts, while non-Hispanic, sexual minority males are more likely to consider switching for other reasons, which could not be assessed in the current study. Future research should assess the motivation and reasons for considering switching from smoking to ONP use by sex, particularly regarding different demographic subgroups.

We also observed differences in associated factors by past history of smoking quit attempts, showing that male gender and current nicotine pouch use was associated with switching regardless of past-year quit attempts; but sexual minority identity and high levels of internalizing tendencies for adult who currently smoke was associated with considering switching only among those who have tried to quit cigarette smoking in the past 12 months, suggesting that they might consider switching ONP use based on their past failed smoking cessation attempts. Given that smoking cessation might be challenging for these sexual minority and adults with higher internalizing tendency subgroups (U.S. Department of Health and Human Services., 2020), ONP use might be their “last resort” for smoking cessation. Future studies should validate our findings and qualitatively assess their motivation for considering switching from cigarettes to ONPs to inform tailored smoking cessation for subgroups.

Our finding that those who had already tried other evidence-based smoking cessation strategies were more likely to consider switching to ONPs compared to those who did not try to quit cigarette smoking supports the idea that ONPs are being perceived by some as cessation aids (Lee et al., 2025, Tosakoon et al., 2023). Also, adults who smoke are and who are currently using ONPs report higher levels of intention to quit their cigarette smoking within the next months (Azagba et al., 2025). This might suggest that harm reduction approaches could be considered for people who have tried to use other methods to quit cigarette smoking.

Emerging evidence shows the effectiveness of ONPs for smoking cessation. A recent Cochrane systematic review and meta-analysis analyzed 4 small studies (i.e., n < 150 for each) and observed limited evidence and certainty on the ONP use for cigarette smoking cessation and reduction currently, even though smoking abstinence may be slightly higher among people who were randomized to ONP use groups (Hartmann-Boyce et al., 2025). Another systematic review and meta-analysis by Heshmati and colleagues analyzed seven randomized controlled trials and crossover trials and observed ONP products might show significantly lower risk of harm than cigarette smoking (Heshmati et al., 2025a). As many clinical trials are ongoing (Pant and Anderer, 2026), more research is needed to examine whether ONP use is actually effective for smoking cessation and a comparative study between ONP use and other methods on smoking cessation among adults who smoke.

### 4.1 Limitations

This study has several limitations. First, as it was an observational secondary analysis of a cross-sectional dataset, causal and temporal relationships between each factor and the outcome could not be established. Note that this study examined the consideration of switching from smoking to ONP use and asked about current ONP use at the same time point, as this is the first wave of the study that has assessed these behaviors; thus, the data are limited to cross-sectional analyses. Future studies should examine whether intention was predictive of future transition behaviors. Second, there may have been unmeasured confounders significantly associated with considering switching to ONPs. Future studies should assess other factors, particularly those related to ONPs, such as product characteristics (e.g., flavor and price), risk perception of ONPs, and exposure to ONP claims and marketing (Hardin et al., 2025; Long et al., 2023). Third, the overall outcome event is low even though the models were converged. To alleviate this concern, we ran two sensitivity analyses with a parsimonious model with careful covariate selection and a modified Poisson regression model with a robust error variance. Given the growing popularity of nicotine pouch use and products, we expect a larger sample size for the future,

## 5 Conclusions

This study identified factors associated with considering switching to nicotine pouches among U.S. adults who smoke, including being male, non-Hispanic, a sexual minority, current nicotine pouch use, and having previously attempted to quit cigarette smoking. We also observed differences in associated factors by sex and by past history of quit smoking attempts. These findings can inform future research on the effects of ONP use on smoking cessation among adults who smoke, particularly within these subgroups. Further, future and ongoing randomized controlled trials examining the impact of ONP use on smoking cessation may consider stratifying analyses or participant recruitment by these subgroups. Further, potential messaging campaigns or counseling approaches that could encourage full switching from smoking to ONP use among these populations, relying on communicating the relative risk of tobacco products. Policymakers and clinicians should also consider these subgroups when developing and implementing ONP-related policies and guidelines. While any nicotine use is considered to be harmful for adolescents, and a careful and extensive review of safety and long term health consequences of ONP use (e.g., preventing or reducing adverse events) is still warranted, the potential for ONPs to reduce harm from smoking in adults should be encouraged.

## CRediT authorship contribution statement

**Rachel N. Cassidy:** Writing – review & editing, Investigation. **Juhan Lee:** Writing – review & editing, Writing – original draft, Investigation, Formal analysis, Conceptualization.

## Author disclosures

None

## Funding

None

## Declaration of Competing Interest

None.

## Footnotes

Appendix A Supplementary data associated with this article can be found in the online version at doi:10.1016/j.dadr.2026.100420.
