# Supplementary material for: Factors associated with considering switching to nicotine pouches among US adults who smoke
Source: Drug Alcohol Depend Rep. 2026 Mar 6;19:100420. doi: 10.1016/j.dadr.2026.100420 (PMC12995810; doi:10.1016/j.dadr.2026.100420)
Supplement: Supplementary file 1 — Supplementary material [file mmc1.docx]

**Supplemental Table 1**. Results of the multivariable binomial logistic regression model on having considered switching from cigarette smoking to nicotine pouches (such as Zyn, On! or Velo) by *a priori* sociodemographic and behavioral predictors, stratified by current ONP use status

|  | **Outcome: Having considered switching from cigarette smoking to nicotine pouches (such as Zyn, On! or Velo)** | | | |
| --- | --- | --- | --- | --- |
|  | **Among no past-30-day ONP use (N=6,178)** | | **Among past-30-day ONP use (N=128)** | |
|  | Adjusted Odds Ratio (95% CI) | P-value | Adjusted Odds Ratio (95% CI) | P-value |
| **Age, years** |  |  |  |  |
| 35 or more | Reference |  | Reference |  |
| 18-34 | 1.48 (0.87, 2.51) | 0.151 | 0.78 (0.13, 4.59) | 0.782 |
| **Sex** |  |  |  |  |
| Female | Reference |  | Reference |  |
| Male | **2.74 (1.37, 5.48)** | **0.005** | 2.19 (0.23, 20.78) | 0.491 |
| **Race** |  |  |  |  |
| White | Reference |  | Reference |  |
| Black | 0.95 (0.42, 2.13) | 0.900 | 1.94 (0.11, 33.05) | 0.644 |
| Others | 0.72 (0.26, 1.97) | 0.514 | 0.03 (0.00, 3.81) | 0.157 |
| **Ethnicity** |  |  |  |  |
| Non-Hispanic | 3.00 (0.86, 10.52) | 0.085 | 4.13 (0.23, 73.70) | 0.332 |
| Hispanic | Reference |  | Reference |  |
| **Annual household income** |  |  |  |  |
| Less than $50,000 | Reference |  | Reference |  |
| More than $50,000 | 0.90 (0.41, 1.95) | 0.783 | 2.32 (0.37, 14.35) | 0.362 |
| **Sexual identity** |  |  |  |  |
| Heterosexual | Reference |  | Reference |  |
| LGB+ | 2.59 (0.97, 6.91) | 0.057 | 3.30 (0.19, 56.17) | 0.405 |
| **Region** |  |  |  |  |
| Northeast | Reference |  | Reference |  |
| Midwest | 1.05 (0.32, 3.43) | 0.932 | 3.09 (0.20, 48.68) | 0.418 |
| South | 1.56 (0.51, 4.76) | 0.435 | 0.22 (0.01, 4.10) | 0.304 |
| West | 1.29 (0.35, 4.82) | 0.700 | 2.87 (0.20, 42.05) | 0.437 |
| **Internalizing tendencies** |  |  |  |  |
| Low | Reference |  | Reference |  |
| Moderate | 1.66 (0.70, 3.95) | 0.247 | 1.75 (0.18, 16.87) | 0.625 |
| High | 1.76 (0.61, 5.07) | 0.294 | 5.76 (0.34, 97.26) | 0.222 |
| **Externalizing tendencies** |  |  |  |  |
| Low | Reference |  | Reference |  |
| Moderate | 0.99 (0.44, 2.25) | 0.984 | 4.09 (0.43, 38.52) | 0.216 |
| High | 1.45 (0.49, 4.23) | 0.496 | 0.25 (0.01, 7.07) | 0.415 |
| **Past-30-day e-cigarette use** |  |  |  |  |
| No | Reference |  | Reference |  |
| Yes | 1.28 (0.65, 2.55) | 0.472 | 1.42 (0.38, 5.26) | 0.599 |
| **Past-30-day smokeless/snus use** |  |  |  |  |
| No | Reference |  | Reference |  |
| Yes | 1.87 (0.53, 6.58) | 0.324 | 0.87 (0.14, 5.27) | 0.877 |
| **Days of cigarette smoking in the past 30 days** |  |  |  |  |
| Continuous (ranged 0-30) | 1.02 (0.99, 1.05) | 0.194 | 0.95 (0.89, 1.02) | 0.172 |
| **Past-12-month having tried to quit cigarette smoking** |  |  |  |  |
| No | Reference |  | Reference |  |
| Yes | **2.30 (1.15, 4.60)** | **0.018** | 2.08 (0.30, 14.63) | 0.457 |
| **Harm perception toward nicotine** |  |  |  |  |
| Treated as continuous (from “Not at all harmful” to “Extremely harmful”) | 1.05 (0.80, 1.38) | 0.737 | 0.62 (0.26, 1.45) | 0.264 |

**Supplemental Table 2.** Results of the multivariable binomial logistic regression model on having considered switching from cigarette smoking to nicotine pouches (such as Zyn, On! or Velo), with a 3-level variable with quit attempt and whether used evidence-based smoking cessation strategies.

|  | **Outcome: Having considered switching from cigarette smoking to nicotine pouches (such as Zyn, On! or Velo)** | |
| --- | --- | --- |
|  | Adjusted Odds Ratio (95% CI) | P-value |
| **Age, years** |  |  |
| 35 or more | Reference |  |
| 18-34 | 1.61 (0.99, 2.62) | 0.056 |
| **Sex** |  |  |
| Female | Reference |  |
| Male | **2.75 (1.45, 5.21)** | **0.002** |
| **Race** |  |  |
| White | Reference |  |
| Black | 1.05 (0.48, 2.26) | 0.907 |
| Others | 0.50 (0.18, 1.41) | 0.187 |
| **Ethnicity** |  |  |
| Non-Hispanic | **3.23 (1.23, 8.48)** | **0.018** |
| Hispanic | Reference |  |
| **Annual household income** |  |  |
| Less than $50,000 | Reference |  |
| More than $50,000 | 1.17 (0.63, 2.15) | 0.616 |
| **Sexual identity** |  |  |
| Heterosexual | Reference |  |
| LGB+ | **2.46 (1.03, 5.88)** | **0.042** |
| **Region** |  |  |
| Northeast | Reference |  |
| Midwest | 1.20 (0.47, 3.10) | 0.697 |
| South | 1.11 (0.41, 3.01) | 0.835 |
| West | 1.46 (0.56, 3.79) | 0.438 |
| **Internalizing tendencies** |  |  |
| Low | Reference |  |
| Moderate | 1.76 (0.85, 3.65) | 0.130 |
| High | 1.85 (0.80, 4.31) | 0.151 |
| **Externalizing tendencies** |  |  |
| Low | Reference |  |
| Moderate | 1.16 (0.59, 2.27) | 0.659 |
| High | 0.98 (0.38, 2.56) | 0.969 |
| **Past-30-day e-cigarette use** |  |  |
| No | Reference |  |
| Yes | 1.34 (0.79, 2.27) | 0.274 |
| **Past-30-day smokeless/snus use** |  |  |
| No | Reference |  |
| Yes | 1.33 (0.59, 3.02) | 0.489 |
| **Past-30-day nicotine pouch use** |  |  |
| No | Reference |  |
| Yes | **28.61 (12.23, 66.91)** | **<0.001** |
| **Days of cigarette smoking in the past 30 days** |  |  |
| Continuous (ranged 0-30) | 1.00 (0.98, 1.03) | 0.790 |
| **Past-12-month having tried to quit cigarette smoking** |  |  |
| Did not have tried to quit cigarette smoking | Reference |  |
| Have tried to quit cigarette smoking, but did not use evidence-based smoking cessation strategies | 1.67 (0.83, 3.37) | 0.150 |
| Have tried to quit cigarette smoking, and used evidence-based smoking cessation strategies | **3.10 (1.30, 7.36)** | **0.011** |
| **Harm perception toward nicotine** |  |  |
| Treated as continuous (from “Not at all harmful” to “Extremely harmful”) | 0.97 (0.78, 1.21) | 0.781 |

“Evidence-based smoking cessation strategies” include counseling, a telephone help line or quit line, books, pamphlets, videos, a quit tobacco clinic, class, or support group, or an internet or web-based program, a nicotine patch, gum, inhaler, nasal spray or lozenge, Chantix, varenicline, Wellbutrin, Zyban, or bupropion

**Supplemental Table 3.** Results of the multivariable binomial logistic regression model on having considered switching from cigarette smoking to nicotine pouches (such as Zyn, On! or Velo) by a priori sociodemographic and behavioral predictors, including only factors with p<0.2 in their bivariate associations

|  | **Outcome: Having considered switching from cigarette smoking to nicotine pouches (such as Zyn, On! or Velo)** | |
| --- | --- | --- |
|  | Adjusted Odds Ratio (95% CI) | P-value |
| **Age, years** |  |  |
| 35 or more | Reference |  |
| 18-34 | 1.50 (0.93, 2.43) | 0.098 |
| **Sex** |  |  |
| Female | Reference |  |
| Male | **2.89 (1.60, 5.23)** | **0.001** |
| **Race** |  |  |
| White | Reference |  |
| Black | 0.98 (0.50, 1.89) | 0.943 |
| Others | 0.52 (0.19, 1.37) | 0.184 |
| **Ethnicity** |  |  |
| Non-Hispanic | **3.31 (1.27, 8.57)** | **0.014** |
| Hispanic | Reference |  |
| **Sexual identity** |  |  |
| Heterosexual | Reference |  |
| LGB+ | **2.50 (1.08, 5.76)** | **0.032** |
| **Internalizing tendencies** |  |  |
| Low | Reference |  |
| Moderate | 1.82 (0.88, 3.77) | 0.104 |
| High | 1.93 (0.86, 4.32) | 0.108 |
| **Externalizing tendencies** |  |  |
| Low | Reference |  |
| Moderate | 1.15 (0.59, 2.25) | 0.670 |
| High | 1.06 (0.43, 2.65) | 0.899 |
| **Past-30-day e-cigarette use** |  |  |
| No | Reference |  |
| Yes | 1.33 (0.78, 2.26) | 0.297 |
| **Past-30-day smokeless/snus use** |  |  |
| No | Reference |  |
| Yes | 1.32 (0.58, 2.97) | 0.506 |
| **Past-30-day nicotine pouch use** |  |  |
| No | Reference |  |
| Yes | **32.42 (14.26, 73.71)** | **<0.001** |
| **Days of cigarette smoking in the past 30 days** |  |  |
| Continuous (ranged 0-30) | 1.00 (0.98, 1.02) | 0.808 |
| **Past-12-month having tried to quit cigarette smoking** |  |  |
| No | Reference |  |
| Yes | **1.95 (1.07, 3.58)** | **0.030** |

**Supplemental Table 4**. Results of the multivariable modified Poisson regression model with a robust error variance on having considered switching from cigarette smoking to nicotine pouches (such as Zyn, On! or Velo) by a priori sociodemographic and behavioral predictors, to reduce the potential bias from the low number of outcome events.

|  | **Outcome: Having considered switching from cigarette smoking to nicotine pouches (such as Zyn, On! or Velo)** | |
| --- | --- | --- |
|  | Adjusted Prevalence Ratio (95% CI) | P-value |
| **Age, years** |  |  |
| 35 or more | Reference |  |
| 18-34 | 1.52 (1.00, 2.32) | 0.051 |
| **Sex** |  |  |
| Female | Reference |  |
| Male | **2.48 (1.39, 4.43)** | **0.002** |
| **Race** |  |  |
| White | Reference |  |
| Black | 0.93 (0.48, 1.83) | 0.837 |
| Others | 0.55 (0.22, 1.37) | 0.196 |
| **Ethnicity** |  |  |
| Non-Hispanic | **2.81 (1.18, 6.67)** | **0.020** |
| Hispanic | Reference |  |
| **Annual household income** |  |  |
| Less than $50,000 | Reference |  |
| More than $50,000 | 1.12 (0.68, 1.87) | 0.651 |
| **Sexual identity** |  |  |
| Heterosexual | Reference |  |
| LGB+ | **2.21 (1.07, 4.57)** | **0.033** |
| **Region** |  |  |
| Northeast | Reference |  |
| Midwest | 1.21 (0.52, 2.79) | 0.658 |
| South | 1.18 (0.48, 2.91) | 0.716 |
| West | 1.36 (0.58, 3.18) | 0.474 |
| **Internalizing tendencies** |  |  |
| Low | Reference |  |
| Moderate | 1.75 (0.95, 3.24) | 0.073 |
| High | 1.84 (0.94, 3.61) | 0.075 |
| **Externalizing tendencies** |  |  |
| Low | Reference |  |
| Moderate | 1.11 (0.63, 1.94) | 0.711 |
| High | 0.81 (0.37, 1.78) | 0.590 |
| **Past-30-day e-cigarette use** |  |  |
| No | Reference |  |
| Yes | 1.22 (0.76, 1.96) | 0.412 |
| **Past-30-day smokeless/snus use** |  |  |
| No | Reference |  |
| Yes | 1.21 (0.65, 2.26) | 0.551 |
| **Past-30-day nicotine pouch use** |  |  |
| No | Reference |  |
| Yes | **16.92 (8.56, 33.47)** | **<0.001** |
| **Days of cigarette smoking in the past 30 days** |  |  |
| Continuous (ranged 0-30) | 1.00 (0.99, 1.02) | 0.673 |
| **Past-12-month having tried to quit cigarette smoking** |  |  |
| No | Reference |  |
| Yes | **1.89 (1.11, 3.22)** | **0.020** |
| **Harm perception toward nicotine** |  |  |
| Treated as continuous (from “Not at all harmful” to “Extremely harmful”) | 0.98 (0.81, 1.18) | 0.800 |
